# Heyde Syndrome Complicated by Essential Thrombocythemia: A Case Report

**DOI:** 10.7759/cureus.34905

**Published:** 2023-02-12

**Authors:** Motoaki Imawaka, Yudai Tanaka, Tsuyoshi Mishiro, Chiaki Sano, Ryuichi Ohta

**Affiliations:** 1 Family Medicine, Shimane University Medical School, Izumo, JPN; 2 Commnity Care, Unnan City Hospital, Unnan, JPN; 3 Internal Medicine, Unnan City Hospital, Unnan, JPN; 4 Community Medicine Management, Shimane University Faculty of Medicine, Izumo, JPN; 5 Communiy Care, Unnan City Hospital, Unnan, JPN

**Keywords:** family medicine, rural hospital, general medicine, von willebrand disease, primary thrombocytosis, heyde syndrome, gastrointestinal bleeding, aortic stenosis

## Abstract

Heyde syndrome is a multisystem disorder characterized by the triad of aortic stenosis (AS), gastrointestinal bleeding, and acquired von Willebrand syndrome. Age-related degeneration is the most common cause of aortic stenosis and is frequently encountered in today's aging society. Approximately 20% of patients with severe aortic stenosis have Heyde syndrome. We encountered an older patient with primary thrombocytosis who was brought to a rural community hospital with bloody stools and was diagnosed with bleeding from an intestinal arteriovenous malformation. A final diagnosis of Heyde syndrome was made based on the presence of severe aortic stenosis and the presence of schistocytes in peripheral blood smears. Valvular diseases can complicate chronic hematological diseases. When the rapid progression of anemia and segmented red blood cells in the peripheral blood are observed in patients with severe aortic stenosis, Heyde syndrome should be considered based on peripheral blood smears and clinical course.

## Introduction

Heyde syndrome is a syndrome of severe aortic stenosis complicated by gastrointestinal bleeding [1]. In an aging society, the prevalence of aortic stenosis increases and, if severe, the likelihood of developing Heyde syndrome increases [1]. In addition, complications of chronic hematologic diseases may increase the incidence of Heyde syndrome. Chronic hematologic diseases increase the fragility of blood cell components, leading to the progressive destruction of blood cells at the site of aortic stenosis [2]. In this process, further destruction of large multimers of von Willebrand factor may lead to coagulation disorders and gastrointestinal bleeding, such as Heyde syndrome [3, 4]. We report a case of Hyde syndrome in an 82-year-old woman with essential thrombocythemia, whose chief complaint was lower intestinal bleeding. The patient had persistent anemia that could not be explained by essential thrombocythemia alone. Through this case report, we discuss how to manage a potentially fatal disease, such as Heyde syndrome, while appropriately managing the general condition of older patients.

## Case presentation

An 82-year-old woman presented to a rural community hospital with a chief complaint of bloody stools. On the day of admission, her daughter noticed bloody stools and took the patient to the hospital. She had a medical history of essential thrombocythemia from a bone marrow biopsy 10 years ago, chronic renal failure with renal anemia, Alzheimer's disease, and hypertension. Other medical history included spinal canal stenosis. She was taking aspirin 100 mg, famotidine 20 mg, and spironolactone 50 mg. 

On admission, the patient’s vital signs were as follows: blood pressure, 125/71 mmHg; pulse, 87 beats/min, temperature 37.2°C, respiratory rate, 19 breaths/min; and SpO2 99%. Eyelid conjunctival pallor, external jugular vein distension, internal jugular vein pulsation, a cardiac systolic murmur radiating to both sides of the neck, and leg edema were observed. A digital rectal examination revealed black stools. Laboratory data showed a hemoglobin (Hb) level of 4.3 g/dL, erythrocyte count of 1.37 107/μL, reticulocyte count of 5.2%, lactate dehydrogenase (LDH) of 627 U/L, blood urea nitrogen of 38.8 mg/dl, and serum creatinine of 1.13 mg/dL (Table 1).

**Table 1:** Initial laboratory data of the patient PT, prothrombin time; INR, international normalized ratio; APTT, activated partial thromboplastin time; eGFR, estimated glomerular filtration rate; ADAMTS, A Disintegrin and Metalloproteinase with Thrombospondin Motifs; SARS-CoV-2, severe acute respiratory syndrome coronavirus 2; Cl, Chloride

Parameters	Level	Reference
White blood cells	6.30	3.5–9.1 × 10^3^/μL
Neutrophils	61.3	44.0–72.0%
Lymphocytes	15.6	18.0–59.0%
Monocytes	20.7	0.0–12.0%
Eosinophils	1.2	0.0–10.0%
Basophils	1.2	0.0–3.0%
Red blood cells	1.37	3.76–5.50 × 10^6^/μL
Hemoglobin	4.3	11.3–15.2 g/dL
Hematocrit	12.5	33.4–44.9%
Mean corpuscular volume	91.1	79.0–100.0 fl
Platelets	35.5	13.0–36.9 × 10^4^/μL
Total protein	5.5	6.5–8.3 g/dL
Albumin	3.0	3.8–5.3 g/dL
Total bilirubin	0.4	0.2–1.2 mg/dL
Aspartate aminotransferase	23	8–38 IU/L
Alanine aminotransferase	10	4–43 IU/L
γ-Glutamyl transpeptidase	23	<48 IU/L
Lactate dehydrogenase	627	121–245 U/L
Blood urea nitrogen	38.8	8–20 mg/dL
Creatinine	1.13	0.40–1.10 mg/dL
eGFR	35.4	>60.0 mL/min/L
Serum sodium	137	135–150 mEq/L
Serum potassium	4.8	3.5–5.3 mEq/L
Serum Cl	106	98–110 mEq/L
Serum glucose	123	70–110 mg/dL
Creatinine kinase	1782	56–244 U/L
C-reactive protein	1.74	<0.30 mg/dL
PT	80.3	70–130%
PT-INR	10.99	
APTT	27.9	25–40 s
Fibrinogen degradation products	2.8	<5 μg/mL
SARS-CoV-2	Negative	Negative
Urine test		
Leukocyte	Negative	Negative
Nitrite	Negative	Negative
Protein	Negative	Negative
Glucose	Negative	Negative
Urobilinogen	(1+)	Negative
Bilirubin	Negative	Negative
Ketone	Negative	Negative
Blood	Negative	Negative

**Figure 1:**
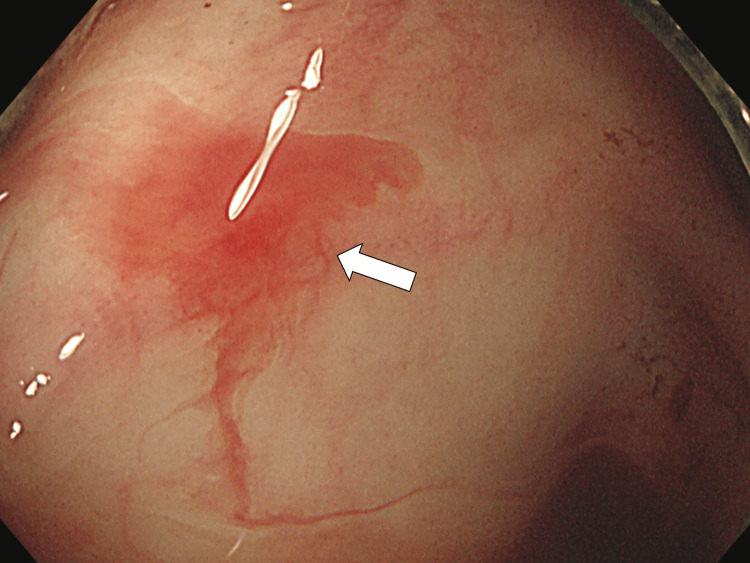
Emergency colonoscopy clarifying that the source of bleeding was an arteriovenous malformation (white arrow)

Contrast-enhanced computed tomography showed high density in the lumen of the ascending colon, indicating active bleeding. An urgent lower gastrointestinal endoscopy was performed, which revealed that the source was an intestinal arteriovenous malformation (Figure 1).

Analysis of the von Willebrand factor showed that the MEDIUM and SMALL multimers were positive, and the LARGE multimer was negative, exhibiting an abnormal multimer distribution. We performed echocardiography showing severe aortic stenosis (aortic valve area, 0.7 cm^2^; aortic flow velocity, 4.1 m/second). With normal coagulation factor levels and an abnormal result of von Willebrand factor analysis, we suspected acquired von Willebrand disease due to essential thrombocythemia, and this, along with aortic stenosis and gastrointestinal bleeding, formed the triad of Heyde syndrome. [3]. 　

**Figure 2:**
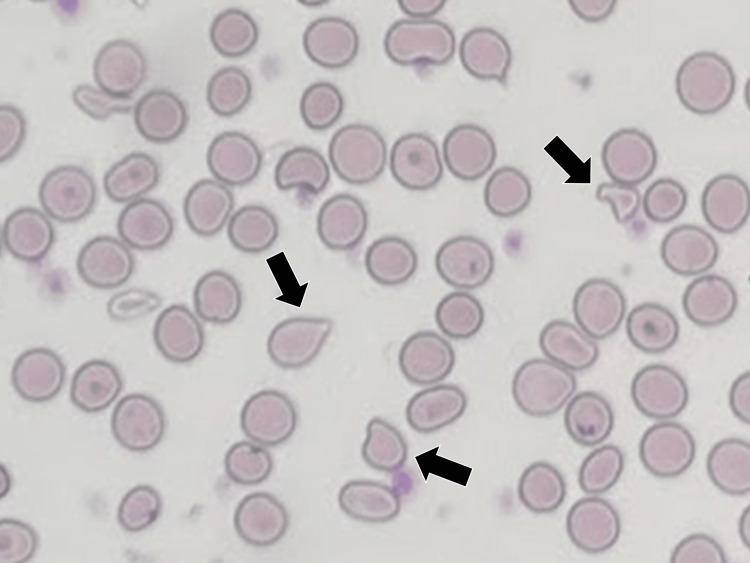
Fragmented red blood cells in the patient’s blood smear (black arrows)

On the second day of hospitalization, the patient’s Hb increased to 5.6 g/dL, and after two more units of blood cell transfusions were administered, it further improved to 7.8 g/dL on the third day. Examination of the peripheral blood smear showed anisocytosis, nucleated erythrocytes, juvenile granulocytes, acanthocytes, and tear-drop erythrocytes (Figure 2).

**Figure 3:**
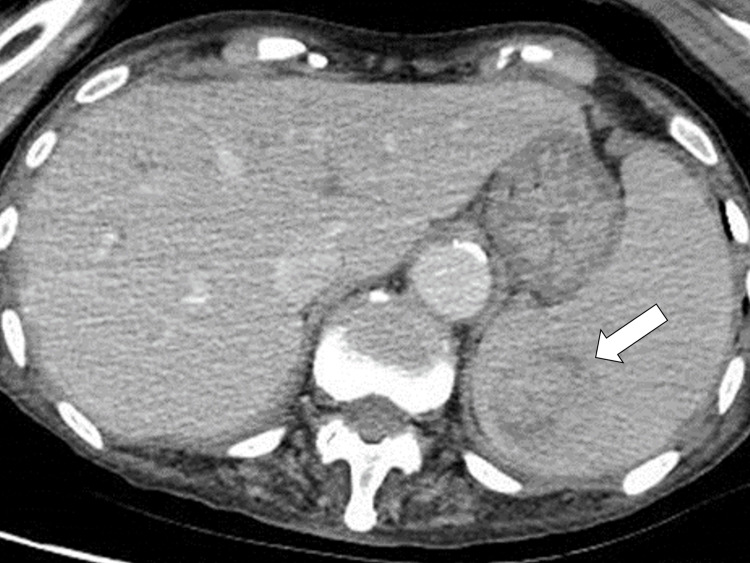
Computer tomography showing a splenic abscess (white arrow).

The patient had a mild fever on admission, which increased to 40.0 °C on the sixth day of hospitalization, with mild tenderness and tapping pain in the left upper abdomen. Based on abdominal computed tomography findings, we suspected a splenic abscess and considered the possibility of bacterial translocation (Figure 3).

The patient was started on piperacillin/tazobactam (13.5 g/day) as empirical therapy. In addition to infection, tumor and drug-induced fever were also considered differential diagnoses. The fever subsided and the blood cultures were negative. Piperacillin/tazobactam was discontinued on the 12th day of hospitalization. On the 23rd day, the anemia and fever due to gastrointestinal hemorrhage improved, and the patient was discharged.

## Discussion

This case shows that the large multimers of von Willebrand factor may be mechanically disrupted by aortic valve stenosis, resulting in coagulation disorder and bleeding from an intestinal arteriovenous malformation [1]. Here, we discuss the difficulty in diagnosing Heyde syndrome in patients with chronic hematologic diseases and the optimal medical treatment for this syndrome.

The incidence of Heyde syndrome may increase among patients with hematologic diseases because of the fragility of their blood cells; therefore, clinicians should consider this disease among older patients with hematologic diseases [1, 5]. The patient, in this case, had an extreme elevation of LDH, abnormal renal function, and anemia that could not be explained by essential thrombocythemia alone. Chronic hematological diseases, such as essential thrombocythemia and polycythemia, may increase the vulnerability of the blood cell components. Population aging may also increase the vulnerability of blood cell components [6]. In addition, aging is often associated with increased calcification and sclerosis of the aortic valve [7]. Therefore, older patients with chronic hematologic diseases may be at a higher risk of Heyde syndrome. Therefore, clinicians should consider this disease in older patients with aortic stenosis. 

As the aging population makes invasive treatment of Heyde syndrome more complex, various treatment options need to be considered. Aortic valve replacement is regarded as the most promising treatment for Heyde syndrome as a long-term treatment for bleeding [8]. However, its invasive nature may make it risky to administer postoperative antiplatelet and anticoagulant medication. There are reports of cases in which the disease was controlled by the administration of bevacizumab, an angiogenic therapy, and octreotide, which lowers venous pressure in the portal system and treats bleeding from angiodysplasia [5, 9]. These treatments can reduce the need for blood transfusions and decrease the risk of bleeding. Recently, transcatheter arterial valve implantation (TAVI), in which aortic valve replacement is performed by catheterization, has become common in elderly patients with aortic valve disease [10]. Considering TAVI in older patients with severe aortic stenosis in uncomplicated settings may improve the quality of life. Aortic stenosis is changing from a disease that causes sudden death to a chronic condition that causes various complaints. Prompt detection and treatment of aortic stenosis are essential for preventing various complaints among older patients.

When a patient presents with abnormal LDH levels, prolonged renal dysfunction, and anemia that cannot be explained by the primary disease, as in our case, the possibility of Heyde syndrome should be considered. As a general practitioner, it is necessary to consider systemic findings, arrive at an early diagnosis, and initiate early treatment of a severe condition by early detection of angiodysplasia in the gastrointestinal tract [11]. An aging society makes it difficult for organ-specific specialists to treat patients with multiple coexisting diseases. It is, therefore, necessary for general practice physicians, as system specialists, to understand each patient's condition comprehensively, view the patient as a system, and manage all of their conditions [11, 12]. General physicians as system-specific specialists can treat patients with multiple coexisting diseases, such as our patients, with circulatory, hematological, and intestinal abnormalities in a multidisciplinary manner. System-focused approaches to general medicine can help increase the possibility of early detection of gastrointestinal vascular dysplasia.

## Conclusions

The development and progression of aortic stenosis in older patients with hematologic comorbidities may lead to Heyde syndrome. The coexistence of multiple diseases in the elderly is becoming increasingly more common as the population ages. As systemic specialists, general practice physicians should manage the general conditions of older patients and provide continuous care in smooth collaboration with organ-specific specialists.
